# Optimal Data Collection Time in LoRa Networks—A Time-Slotted Approach

**DOI:** 10.3390/s21041193

**Published:** 2021-02-08

**Authors:** Dimitrios Zorbas, Christelle Caillouet, Khaled Abdelfadeel Hassan, Dirk Pesch

**Affiliations:** 1 Tyndall National Institute, University College Cork, T12R5CP Cork, Ireland; dimzorbas@ieee.org; 2 School of Engineering and Digital Sciences, Nazarbayev University, Nur-Sultan 010000, Kazakhstan; 3 Université Côte d’Azur, CNRS, I3S, Inria, 06902 Sophia Antipolis, France; christelle.caillouet@univ-cotedazur.fr; 4 School of Computer Science and IT, University College Cork, T12R5CP Cork, Ireland; khaled.abdelfadeel@ieee.org

**Keywords:** LoRa, scheduling, resource allocation

## Abstract

LoRa is a low-power and long range radio communication technology designed for low-power Internet of Things devices. These devices are often deployed in remote areas where the end-to-end connectivity provided through one or more gateways may be limited. In this paper, we examine the case where the gateway is not available at all times. As a consequence, the sensing data need to be buffered locally and transmitted as soon as a gateway becomes available. However, due to the Aloha-style transmission policy of current LoRa-based standards, such as the LoRaWAN, delivering a large number of packets in a short period of time by a large number of nodes becomes impossible. To avoid bursts of collisions and expedite data collection, we propose a time-slotted transmission scheduling mechanism. We formulate the data scheduling optimisation problem, taking into account LoRa characteristics, and compare its performance to low complexity heuristics. Moreover, we conduct a set of simulations to show the benefits of synchronous communications on the data collection time and the network performance. The results show that the data collection can reliably be achieved at least 10 times faster compared to an Aloha-based approach for networks with 100 or more nodes. We also develop a proof-of-concept to assess the overhead cost of communicating the schedule to the nodes and we present experimental results.

## 1. Introduction

LoRa is a widely used long range communication technology for the Internet of Things (IoT). It uses a proprietary spread spectrum modulation currently owned by Semtech [1]. It can trade data rate with coverage using multiple Spreading Factors (SF) which typically range from 7 to 12. Depending on the SF value, the channel bandwidth (BW) as well as other modulation characteristics, LoRa can achieve a data rate of up to approximately 22 kbps. The higher the SF and the lower the channel bandwidth, the lower the data rate and, thus, the longer the transmission time. Moreover, parallel transmissions performed on different SFs but on the same channel can be simultaneously decoded by the gateway. LoRa mainly uses license-free sub-gigahertz radio frequency bands (e.g., EU868, US915 [1]) that are subject to radio duty cycle regulations. For example, in the European Union (EU) the nodes are allowed to transmit up to 1% of the time. However, LoRa chips for the 2.4 GHz band have recently been announced by Semtech. All those characteristics in addition to its resistance to potential external interference make LoRa an easy-to-use wireless data collection solution in agriculture, environmental monitoring, and smart-city applications [2].

The main LoRa-based protocol, called LoRaWAN, provides registration, security, and acknowledgement services for LoRa-enabled IoT devices [3]. A LoRaWAN architecture consists of (a) a core—usually wired—network which implements mechanisms to provide the above mentioned services, (b) one or more gateways, and (c) the IoT devices (end-nodes). The end-nodes can reach the gateway(s) in a single hop. In order to achieve high energy-efficiency and low implementation complexity, LoRaWAN does not perform any specific medium access policy for uplink transmissions. In fact, a Pure Aloha transmission policy is adopted [4]. To increase the probability of packet delivery, multiple gateways can be deployed at different locations. However, it has been shown that LoRaWAN presents a high number of collisions and decreased performance in presence of high number of nodes [5,6], especially for confirmed traffic [7].

However, a common problem that occurs in rural areas, as well as in specific deployments such as in mines [8] and forests [9], is the lack of a seamless power source to allow the installation of multiple gateways as well as their interconnection with a core network infrastructure. Even in the case where solar-powered gateways are employed, their availability depends on weather conditions, thus, seamless operation cannot always be guaranteed. In this paper, we assume such a scenario, where the gateway is not available at all times but it can be turned on periodically to carry out the data collection. The problem of gateway unavailability also appears in low orbit LoRa satellites [10]. Since a satellite gateway can provide coverage to only a part of earth’s surface at every time, only a few nodes can successfully upload data in real time.

Since many typical applications in agriculture or in environmental monitoring are not time-critical, a solution to the gateway unavailability problem is to allow sensing nodes to buffer their data in on-board storage and transmit it in bulk when the gateway becomes available. Since the bulk data collection may have to be achieved as fast as possible, nodes tend to transmit their packets with a high rate, which may saturate the channel. In this case, an Aloha-based approach fails to achieve a high delivery ratio due to high number of collisions. However, in the use cases under consideration, we assume that data traffic is somewhat predictable and, thus, efficient scheduling of transmissions in time slots without significant overhead can be achieved in order to reduce or eliminate collisions and reduce the data collection time. Nevertheless, the problem under consideration requires computation and dissemination of the schedule. Both schedule computation and dissemination are two very challenging problems because (a) the schedule has to be as efficient as possible in terms of data collection time, (b) as compact as possible in terms of size, and (c) to be transmitted to the nodes as quickly as possible over low-rate and duty cycle-restricted wireless links.

In our previous work [11], we investigated the gains of using two centralised scheduling algorithms when the number of nodes and their transmission settings (e.g., reliable SFs) are known. The proposed algorithms assign SFs and allocate time slots for transmissions, aiming at generating collision-free schedules with the minimum possible data collection time. The missing parts of this work were that (a) no theoretical background of the Aloha-based data collection methods was provided. (b) The proposed heuristics did not provide any performance guarantees while there was no comparison with the optimal solution even for small-scale scenarios, (c) we neglected the assessment of the transmission of the schedule to nodes which constitutes one of the major problems in LoRa networks scheduling, and finally, (d) the solutions were not experimentally validated. Hence, in this paper, we extend our previous work to add the following contributions:

1. We describe the weaknesses of an Aloha transmission policy for efficient bulk data collection and we formulate the problem of finding the minimum transmission rate to achieve a certain success probability of packet delivery for each node. This transmission rate provides the lower bound of the bulk data collection time in Aloha approaches.
2. We formulate and solve the optimal time-slotted scheduling problem that minimises the data collection time in LoRa-based networks.
3. We implement a prototype of the proposed approach in a proof-of-concept to assess the practicality and performance of the schedule dissemination time and the ability of the nodes to compute the schedule with their own resources.

The rest of the paper is organised as follows: Section 2 surveys recent related works. Section 3 introduces the bulk data collection problem and describes the limitations of the Aloha MAC in this context. In Section 4, we present preliminary information regarding time-slotted communications and in Section 5 we formulate the optimal scheduling optimisation problem. Section 6 presents theoretical and simulation results, while Section 7 describes how a proof of concept was realised along with experimental results from the testbed. Finally, Section 8 concludes the paper and presents ideas for future directions.

## 2. Related Research

The bulk data transmission problem has been investigated in the literature in the context of opportunistic or delay tolerant networks [12]. Different types of communication protocols have been used, from IEEE802.11 [13] to IEEE802.15.4 [14] and RFIDs [15]. In contrast to those technologies, our work focuses on the data collection from devices placed in large geographical areas, thus, a long range protocol such as LoRa is required. Supporting bulk data transmissions over LoRa-based networks is constrained by various limitations and unique characteristics (e.g., duty cycle, multiple pseudo-orthogonal spreading factors, etc.) that do not apply to other conventional networking solutions.

We have recently studied the bulk data collection problem in LoRa-based networks from different perspectives [11,16,17,18]. In [11,16], heuristic approaches were proposed to compute the time-slotted schedule with online and offline schemes, respectively. The offline approach assumes that a set of information such as the number of devices and their SFs is known in advance by the gateway. In contrast, in the online approach [16], only partial knowledge is assumed, allowing this approach to adapt to dynamic changes such as frequent topology changes. Nevertheless, this comes at the expense of higher energy consumption and longer data collection time than the offline approach. Both approaches are centralized, where the schedule is computed at the gateway and disseminated to the end devices. An alternative approach was proposed for Industrial IoT deployments [18], where the authors investigated a decentralized approach, where end devices can autonomously compute their schedule by receiving the minimum possible information from the gateway. This work was designed to meet specific industrial network criteria, such as fast joining times, low power consumption, and low overhead. However, the schedule length tends to increase substantially as the number of devices increases, which greatly increases the data collection time. Finally, the problem of collecting data using LoRaWAN over a single channel was examined in [17]. In this work, optimal SF assignments are proposed so that the average probability of successful packet delivery is maximized. However, this approach is still Aloha-based and does not eliminate collisions. Unlike the previous works, in this paper, we present the complete picture of the bulk data collection problem, we formulate and solve the optimal scheduling problem to minimize the data collection time considering collision-free scheduling and allocation of SFs, and we study practical implementation aspects such as the schedule transmission.

We rely on a time-slotted medium access control (MAC) approach to enable efficient bulk data transmissions in LoRa-based networks. In the literature, similar MAC approaches have been considered as they typically offer higher reliability than that of Pure Aloha [19]. In [20,21,22], various time-slotted MAC strategies are used to mitigate the collisions in dense LoRa/LoRaWAN networks and, thus, improve their scalability. Similar MAC strategies are also considered in [23,24,25,26] to meet the requirements of soft real-time industrial applications. In [27,28,29], time slots are used to enable low-power multi-hop communications in LoRa-based networks to extend the coverage in harsh environments like underground deployments. In addition to that, the Time Slotted Channel Hoping mechanism of the IEEE802.15.4e standard is employed to enable LoRa-based mesh networking [30]. Unlike the profound advantages of the aforementioned time-slotted solutions in terms of reduced packet collisions, they can barely be used for bulk data collection because of the absence of a scheduler to allocate slots to the nodes or because they follow a slotted-Aloha approach which does not eliminate collisions. Since in bulk data collection, the nodes tend to use a high data rate to quickly expedite all transmissions, non collision-free approaches may suffer for long data collection times.

## 3. The Problem of Bulk Data Collection

In this section, we describe some general requirements to achieve bulk data collection and we present a theoretical uplink analysis of an Aloha-based LoRa MAC to show that the average data collection time increases exponentially with the number of nodes.

### 3.1. Requirements

In order to achieve bulk data collection, as the gateway cannot be available at all times, a mechanism to (almost) simultaneously wake up the nodes once the gateway becomes available must exist. A simple way to achieve that is to schedule the data collection process at predefined times. Apparently, due to the clock drift of the nodes, some of them will wake up earlier than others. Taking into account experimental data from our previous work [31], the maximum clock drift does not exceed 100 ppm, which is translated to a few seconds of time assuming that the data collection is executed once per day. However, having in mind that the data collection may take several hours to be completed (see experimental results in Section 6), the waking up waiting cost is negligible compared to the duration of the data collection. The gateway can initiate the data collection process after this short period of time. In the case of non-coordinated transmissions, such as in an Aloha approach, this can happen by simply sending an initialisation packet. However, if the transmissions are time-synchronised, the gateway needs to compute the schedule and send it to the nodes before starting the data collection. As we already mentioned, the computation and the transmission of the schedule are two critical operations that may considerably affect the reliability of the system as well as the data collection time. These two operations are the two main research subjects of the current paper.

Moreover, since the gateway is expected to be online for multiple hours per day, new nodes can periodically wake up and register with the gateway. If the gateway is not present at that time, the node can return to sleep mode and wake up at a future time to try to register again. Since a gateway system consists of multiple transceivers and the registration takes place at a different frequency, registrations do not affect the data collection process. As a consequence, even if a joining node tries to register during the data collection phase, the latter would not be interrupted/interfered by the registration activity. Registrations can also be accelerated by considering a similar process to LoRaWAN’s personalised activation (ABP). In this case, the gateway is aware of the new nodes while the corresponding DevAddr address as well as the security keys can be stored in the devices.

### 3.2. Data Collection Using Pure and Slotted Aloha MAC

In this subsection, we formulate a data collection time minimisation problem, as a function of the minimum required transmission rate and a given success probability of delivery. We show that reliable bulk data collection in Aloha (Pure and Slotted) cannot be achieved in a short amount of time even for a low number of nodes.

In Table 1 we define a set of notations and their meaning to assist in the understanding of remainder of the presentation.

Aloha-based MAC protocols, such as used in LoRaWAN, do not apply any mechanism to coordinate packet transmissions. Indeed, each node transmits its packets whenever data are available and the duty cycle restrictions are met. In this case, some of the packets are delivered while others are lost due to collisions. Specifically, the collision rate depends on this transmission rate. For example, allowing the nodes to transmit in sparse intervals decreases the collisions but prolongs the data collection time. Assuming that all nodes transmit the same amount of packets pkt, the average data collection time is then equal to pkt/θ, where θ is the packet transmission rate.

**Figure 1:**
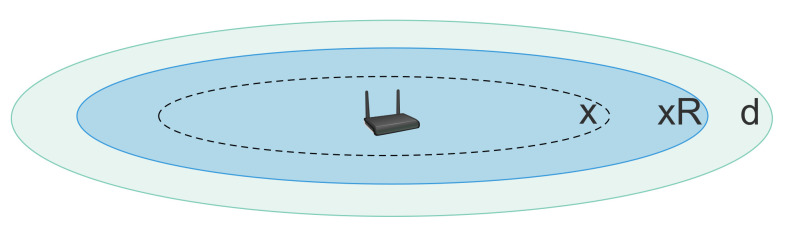
Interfering zone (dark colour) of a node located at distance *x* from the gateway placed at the center of the disk (intra-Spreading Factor (SF) interference).

In order to model an Aloha MAC behaviour for the bulk data collection scenario, we assume that all nodes’ packets are equal in length and their transmissions follow a Poisson distribution with intensity θ packets/s. Assuming the best-case scenario of no inter-SF collisions, transmissions only collide when they overlap in SF, time, and power [6]. Given the analysis provided in [17], if a node at distance *x* from the gateway transmits a packet with SF *f*, the transmission will be successful if it does not overlap with any other packets in the network having the same SF within the transmission time $T_{f}$. Alternatively, due to the capture effect, the packet can be decoded if the received power of other packets with the same SF is less than the current one by at least $P_{\mathrm{thld}}$. As shown in Figure 1, considering a log-distance path loss model, the potential interfering nodes are those whose distance from the gateway is below xR with $R=10^{\frac{P_{\mathrm{thld}}}{10\gamma }}>1$ [17], where γ is the path loss exponent.

Consequently, given a uniform distribution of nodes with SF *f*, the number of potential interfering nodes is $N_{f}\frac{\pi {\left(\min\left(Rx,d\right)\right)}^{2}}{\pi d^{2}}$, where $N_{f}$ is the number of nodes with SF *f* and *d* is the range of the deployment for the given SF. The probability of successful delivery $P_{f}\left(x\right)$ is that within a vulnerability period with duration $2T_{f}$, none of those potential interfering nodes starts a transmission. Hence, we have:

(1) $$\begin{array}{c} P_{f}\left(x\right)=e^{-2T_{f}\theta N_{f}\frac{{\left(\min\left(xR,d\right)\right)}^{2}}{d^{2}}}. \end{array}$$

The shorter the distance *x* to the gateway, the higher the probability $P_{f}\left(x\right)$ of success delivery due to less interferers. The worst case situation appears for nodes that are on the border of the deployment range (i.e., x→d). In this case, all nodes with the same SF are potential interferers and the minimum success probability $P_{f}^{min}$ is then equal to $P_{f}^{min}=e^{-2T_{f}\theta N_{f}}$.

As the transmission rate of the nodes decreases, the success probability increases, but so does the data collection time. This means that there is a certain (maximum) transmission rate $\theta_{max}$ that satisfies a minimum success probability for all the nodes in the network. The question that arises is: how long is the data collection time given this rate and the desired minimum allowed success probability? In order to answer this question, we formulate the following optimisation problem:

(2) $$\begin{array}{cc} & \max\theta ,\\ \mathrm{subject}\;\mathrm{to}: & \end{array}$$

(3) ∑k=ρpktpktkPfmink1−Pfminpkt−k≥Pgiven,

(4) $$\begin{array}{cc} & P_{f}^{min}=e^{-2T_{f}\theta N_{f}}, \end{array}$$

(5) $$\begin{array}{cc} & \theta T_{f}\leq C. \end{array}$$

Equation (3) requires that the probability of “at least ρ packets (i.e., $\rho \in N^{*},\rho \leq pkt$) are successfully delivered” is greater than or equal to a given value $P_{given}$, and $pktN_{f}$ is large enough. Equation (5) guarantees that the duty cycle constraint *C* is not exceeded (if there is no duty cycle restriction then C=1, for example, in 2.4 GHz LoRa). We must note that in presence of a slotted-Aloha protocol, the vulnerability period reduces from 2$T_{f}$ to $T_{f}$. Thus, Equation (4) can be modified accordingly.

**Figure 2:**
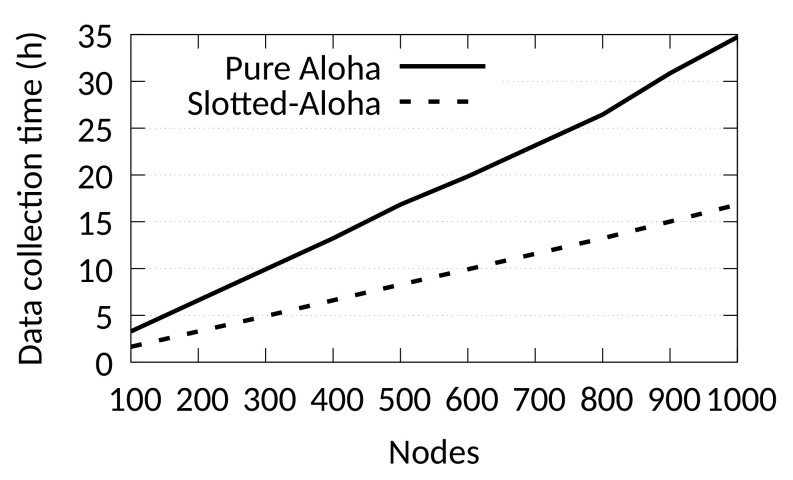
Theoretical average data collection time (pkt/θ) of an Aloha and Slotted-Aloha LoRa MAC for different node populations, pkt = 100, and 100-bytes packet size . At least 90% packet success rate of all the nodes is guaranteed.

The solution of the optimisation problem gives the theoretical upper bound for the transmission rate (i.e., $\theta_{max}$) and, hence, the lower bound for the required data collection time. The example of Figure 2 depicts the theoretical data collection time for a scenario with 100 to 1000 nodes and a minimum transmission success probability of 0.9. All nodes use a 100-byte packet size, transmit 100 packets, and reach the gateway with SF7. We can observe that the data collection may take several hours even for low node numbers. This is because the data collection time depends on the packet rate θ which is computed by solving the aforementioned optimization problem. As the number of nodes increases, the number of collisions increase as well. That means that θ has to be increased considerably in order to make transmissions sparser and, thus, reduce the number of collisions. It is obvious from this analysis that an Aloha MAC (Pure or Slotted) cannot achieve a reliable bulk data collection in a reasonable period of time. We should mention that optimally assigning SFs such that the probability of delivery is maximised, can alleviate the problem of collisions [17]. However, even in this case the data collection time remains very long.

## 4. Towards Time-Slotted LoRa Communications

In this section, we briefly describe the proposed time-slotted LoRa architecture consisting of frames, time slots, and a schedule.

### 4.1. Time Slots, Frames, and Synchronisation

The current work uses the same frame design as TS-LoRa [31]. TS-LoRa is a time-slotted protocol for industrial IoT using LoRa as the physical layer. According to TS-LoRa, the time is divided into repeated frames where each frame consists of a number of time slots. Each time slot accommodates the transmission of one node. Assuming packets of equal size and the fact that the transmission time increases with higher SFs, the length of the time slots is the same only for packets transmitted with the same SF. This allows TS-LoRa to have 6 parallel frames where each frame accommodates transmissions of nodes using the same SF. The frames have a minimum allowed size as this is dictated by the radio duty cycle rules, however, additional slots can be added and expand the frame size in order to accommodate more transmissions per frame. Apparently, the longer the frame, the sparser the transmission rate, and thus, the data collection time.

**Figure 3:**
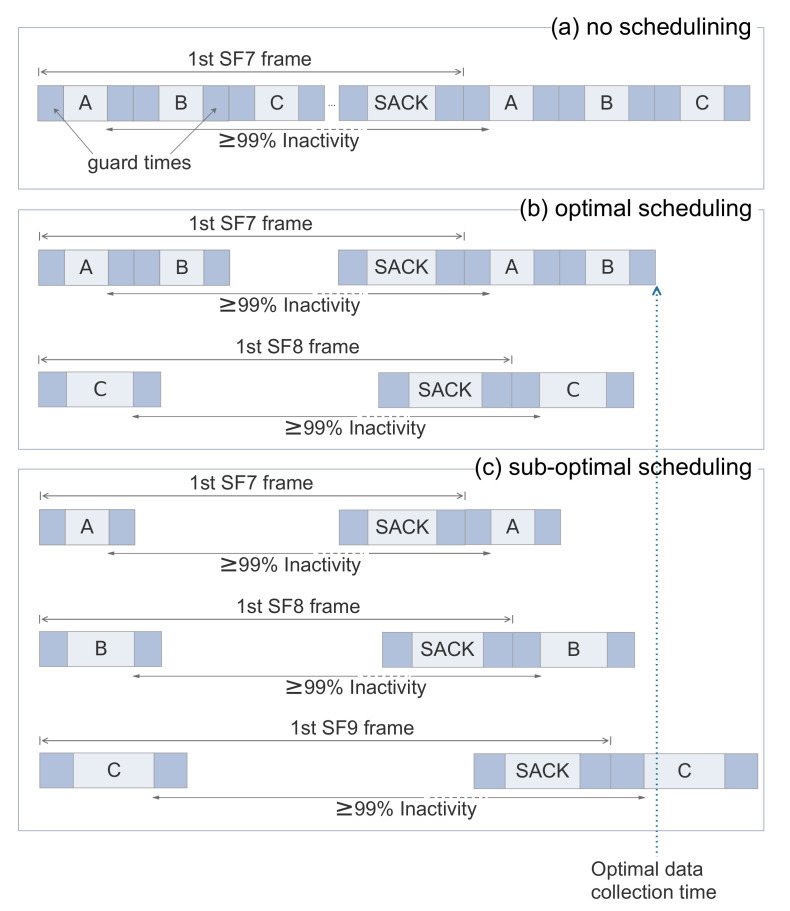
Different slot and SF allocations of three transmissions with the same packet length and SF7: (**a**) no extra scheduling is performed. All nodes are serially placed in slots of the SF7 frame one after the other. This is a sub-optimal solution in terms of data collection time. (**b**) Node C is switched to SF8 frame. This is the optimal solution since it results to minimum data collection time. (**c**) Node B is switched to SF8 frame and Node C to SF9 frame. This is also a sub-optimal solution.

As with all synchronised protocols, the nodes need to be periodically synchronised by the gateway according to a global clock. In order to accommodate the nodes’ clock drift over time, guard times are added between successive time slots to ensure that adjacent transmissions do not overlap. Assuming the propagation delay negligible, the guard time depends on the crystal clock accuracy and on how often the gateway communicates with the nodes to synchronise their clocks. In TS-LoRa, this is performed using a novel mechanism called “SACK” (Synchronisation and ACKnowledgmemts) which merges sync and (optional) ack data into a single downlink packet. The frame size and the guard time are adjusted automatically according to the number of registered nodes. An example of the frame structure is given in Figure 3a. Detailed information of the time-slotted operation and the synchronisation can be found in [31,32].

### 4.2. The Schedule

We call *schedule* a series of frames containing transmissions (slots) from all the nodes in the network. Nodes’ transmission(s) are accommodated in one or more slots. TS-LoRa does not perform any scheduling since the nodes are serially placed into slots as soon as they register with the gateway using the minimum (energy-wise) possible SF. In our current problem, we can still follow the same rationale but this is not enough. This strategy could lead to long data collection times since some frames might be overcrowded. For example, if many nodes are placed close to the gateway, all of them would most likely use SF7 resulting to a large number of slots in that specific frame (See Figure 3a). To tackle that issue, scheduling is performed to find a better arrangement of nodes to slots (other than the serial one) so as to minimise the data collection time without violating duty cycle regulations (whenever they are applied).

Assuming that nodes typically have more than one packet to send, transmissions from each particular node have a minimum periodicity limited by the duty cycle regulations as it is already mentioned. The time slots in between this periodicity can, therefore, be used to schedule transmissions from other nodes. In general, if the airtime (i.e., transmission time) for a SF is $T_{f}$ and the duty cycle is 1%, the minimum period of time between two successive transmissions of the same node is $T_{f}\times 100$ and approximately $N_{f}=\frac{99T_{f}}{T_{f}+2\tau }$ transmissions from other nodes with the same SF can be scheduled in the meantime, where τ is the guard time at the beginning and at the end of a slot. If more nodes need to be assigned, the frame for SF *f* is extended. Moreover, a scheduler follows always a simple rule. A node (or transmission) is allocated to the frame that leads to the earliest completion of the data collection. Figure 3 illustrates such an example of allocation using three nodes with two transmissions each. The optimal solution is to schedule Node C transmissions to a frame with a higher SF since this results in a shorter data collection time (See Figure 3b). If the scheduler allocates each node transmissions to a different frame, this may result to a sub-optimal solution (See Figure 3c).

## 5. The Optimal Data Scheduling Problem

In this section, we formulate the optimal data scheduling problem, that is the optimal arrangement of nodes’ transmissions into slots and frames. This formulation does not take into account acknowledgements since (a) acknowledgments are optional, and (b) the size of the acknowledgements is dynamic and depends on the actual number of nodes and the number of received packets in the frame. Moreover, acknowledgements are natively supported by the frame structure of TS-LoRa in the SACK packet which can be easily added into the following formulation. Nevertheless, for fair compare reasons of the optimal solution with the Aloha (which considerably suffers for the downlink traffic [33]), we skip its formulation. The network is composed of a set of *N* nodes. Depending on the distance of a node *i* from the gateway, a set of possible SFs $F_{i}$ is required and is allocated during the registration of the node with the gateway. For example, if a node can reach the gateway with SF7, all higher SFs will also allow the node to reach the gateway and can be used for different transmissions of the node in the schedule. Each node *i* has an amount of data to transmit, denoted by $pkt_{i}$. Each transmission of *i* can be scheduled using a SF *f* ($f\in F_{i}$) with an associated payload $payload_{f}$. Given *f* and $payload_{f}$, we can calculate the packet transmission time $T_{f}$ [34].

Let F={7,⋯,12} be the set of available spreading factors. To each SF f∈F, we associate a set $T_{f}=\left\{t_{j}^{f},\;1\leq j\leq \Gamma \right\}$ of time slots starting at time *j*, where Γ is the global upper limit in number of slots. These slots have duration $T_{f}+2\tau$ for the given node’s payload and SF, where τ corresponds to the guard time.

We associate a set of forbidden slots $\chi_{t_{j}^{f}}$ to each slot $t_{j}^{f}$. This set contains all the slots $t_{j^{\prime }}^{\prime f^{\prime }}$ of the same SF (i.e., $f^{\prime }=f$), and of other SFs (i.e., $f^{\prime }\neq f$) such that $j\leq j^{\prime }\leq j+100T_{f}$, for 1% duty cycle. If a node uses $t_{j}^{f}$ to transmit data, then the duty cycle restriction forbids the node to use any slot in $\chi_{t_{j}^{f}}$. Apparently, this constraint can be eliminated when no radio duty cycle restrictions exist.

Given the previous input parameters of the problem, we define the following set of binary variables:

$$y_{t_{j}^{f}}^{i}=\left\{\begin{array}{cc} 1 & \mathrm{if}\;\mathrm{node}\;i\;\mathrm{uses}\;\mathrm{slot}\;t_{j}^{f},\\ 0 & \mathrm{otherwise} \end{array}\right.$$

The linear program to optimally schedule bulk data transmissions is defined as follows:

(6) $$\begin{array}{cc} & \min\left(\max_{f\in F,\;t_{j}^{f}\in T_{f}}\left(j+\tau +T_{f}\right)\sum_{i}y_{t_{j}^{f}}^{i}\right)\\ \mathrm{subject}\;\mathrm{to}: & \end{array}$$

(7) $$\begin{array}{cc} & \sum_{i\in N}y_{t_{j}^{f}}^{i}\leq 1,\;\forall f\in F,t_{j}^{f}\in T_{f} \end{array}$$

(8) $$\begin{array}{cc} & \sum_{f\in F\setminus F_{i}}\sum_{t_{j}^{f}\in T_{f}}y_{t_{j}^{f}}^{i}=0,\;\forall i\in N \end{array}$$

(9) $$\begin{array}{cc} & \sum_{f\in F_{i}}\sum_{t_{j}^{f}\in T_{f}}payload_{f}y_{t_{j}^{f}}^{i}\geq pkt_{i},\;\forall i\in N \end{array}$$

(10) $$\begin{array}{cc} & y_{t_{j}^{f}}^{i}+y_{t_{j^{\prime }}^{\prime f^{\prime }}}^{i}\leq 1,\;\forall i\in N,f\in F_{i},t_{j}^{f}\in T_{f},t_{j^{\prime }}^{\prime f^{\prime }}\in \chi_{t_{j}^{f}}. \end{array}$$

The objective function (6) seeks to minimise the length of the longest frame among all SFs. The end of the frame occurs at time $j+\tau +T_{f}$ because the last transmission starts at j+τ and lasts for $T_{f}$ amount of time. To determine which slot is the last one used, we need to multiply $j+\tau +T_{f}$ with the sum over all variables $y_{t_{j}^{f}}^{i}$. Indeed, there can be only one node using a slot, so this sum is either 0 (the slot is not used) or 1 (the slot is used by one node). Thus, the result of $\max_{f\in F,\;t_{j}^{f}\in T_{f}}\left(j+\tau +T_{f}\right)\sum_{i}y_{t_{j}^{f}}^{i}$ gives the time $\left(j+\tau +T_{f}\right)$ of the longest frame. To get a linear equation removing the maximum function, we add the following set of constraints:

(11) $$\left(j+\tau +T_{f}\right)\sum_{i}y_{t_{j}^{f}}^{i}\leq \zeta ,\;\forall f\in F,t_{j}^{f}\in T_{f},$$

where ζ is a continuous variable whose purpose is to keep track of the maximum frame length, which is what we want to minimise in our problem.

Summarising, Equation (7) verifies that at most one node can use a slot. Equation (8) forbids a node to use non-permitted SFs. Equation (9) ensures that all nodes get to send their total amount of data. Finally, Equation (10) verifies the duty cycle restrictions using sets $\chi_{t_{j}^{f}}$ defined above. This last constraint can be eliminated in the case of LoRa operating in the 2.4 GHz ISM band where no radio duty cycle restrictions exist [35].

The hardness of the model stems from the fact that a very large range of slots for all 6 SFs (or 8 for 2.4 GHz LoRa) is evaluated for each possible transmission. This results in a very long sequence of possible iterations. The problem can be alleviated by setting a maximum frame length Γ per SF which is translated to a maximum number of slots to be evaluated. However, we need to define how this value is chosen. For example, allowing too many slots in each frame increases dramatically the number of binary variables of our model (recall that there is a single *y* variable per node and per slot). On the contrary, if we do not reserve enough slots for each SF, especially for SF7 which is the most utilised spreading factor, the linear program can still find a solution with the given slots. In this case, the schedule will be optimal only for the given settings but not globally optimal. Since most of the transmissions are accommodated in the SF7 frame, the value of Γ is selected through a trial and error method so that the frame of SF7 is large enough to find the optimal schedule.

Nevertheless, the linear program still requires to test a high number of variables per transmission, upper-bounded by NΓ|F|, which may limit the practicality of the computational solution to low node numbers only. For deployments with many nodes, time-efficient heuristics such as the *Light* and *Global* heuristics presented in [11] can be used. The two heuristics differ in the way they handle transmissions and how they divide the schedule into frames. The first algorithm, *Light*, schedules only the first transmission for each node and repeats that in subsequent frames. The second approach, *Global*, computes the schedule for all transmissions and, as a consequence, produces a sequence of frames that may differ to each other. As a result, *Global* can compute more efficient schedules than *Light* in terms of data collection time, however, this comes at the expense of a higher computation cost, a larger schedule size, and a higher implementation complexity.

## 6. Evaluation and Discussion of the Results

In this section, simulation and experiments are conducted to evaluate the performance of the optimization model as well as practical issues regarding time-slotted communications in LoRa networks. Due to the high complexity of the model, the LP solver was not able to solve solutions with more than 50 nodes. As a consequence, we divided the simulation results in two sections. Section 6.2 is dedicated to the comparison with the optimal with 10–50 nodes, and Section 6.3 is dedicated to scenarios with a higher number of nodes (100–1000).

### 6.1. Simulation Setup

We consider a square deployment area with a side of 1000 m length and a variable number of nodes that are randomly and uniformly scattered. We also consider a path-loss model that corresponds to a smart city scenario where the signal attenuates fast [6]. We compare the performance of the time-slotted scheduling heuristics, *Light* and *Global* [11], with a Pure Aloha-based approach (denotes the standard LoRaWAN), with a Slotted-Aloha one, as well as with the optimal scheduling solution of Section 5 (for 10 to 50 nodes). For fair comparison (in the case of confirmed transmissions the Aloha approach presents a very high number of collisions), the uplink transmissions are unconfirmed, so no re-transmissions occur (however some experiments with confirmed transmissions are shown in Section 7). In the Aloha-based approaches, the nodes use the minimum reliable SF that allows them to reach the gateway, which actually demonstrates the standard Adaptive Data Rate (ADR) mechanism of LoRaWAN. We allow the nodes to periodically send data with the optimal rate $\theta_{max}$ as derived by solving the optimisation problem in Equations (2)–(5) for both Pure and Slotted-Aloha cases. This optimization problem is solved using exhausting search as with [17]. In this way, we guarantee a packet delivery ratio of at least 90% for all nodes in the network. In Pure Aloha, each node transmits at a constant rate which may vary for a few seconds from transmission to transmission in order to avoid repeated collisions. In Slotted-Aloha, each node chooses randomly a slot to transmit; this slots changes at every transmission also to avoid repeated collisions. In collision-free approaches (i.e., scheduled ones), the maximum allowed data rate as this is dictated by the duty cycle rules and the scheduler is followed.

All scheduling algorithms are implemented in the Perl programming language (the code is available at https://github.com/deltazita/offline-lora (accessed on 3 February 2021)), taking into account the path-loss and fading of the signal and the capture effect at the gateway. The Aloha-based approaches are implemented in a LoRaWAN simulator (LoRaWAN simulator https://github.com/deltazita/LoRaWAN-SIM (accessed on 3 February 2021)) whose effectiveness and correctness have been verified by experiments [17]. The simulator has been adapted to the needs of the bulk data collection and inherits the following capabilities and features from the original simulator:

- Multiple half-duplex gateways
- 1% radio duty cycle for uplink transmissions
- 1% or 10% radio duty cycle for downlink transmissions
- Two receive windows (RX1, RX2) for ACKs and commands
- Non-orthogonal SF transmissions
- Capture effect
- Path-loss signal attenuation model
- Multiple channels (however a single channel was used in this paper)
- Collision handling for both uplink + downlink transmissions
- Node energy consumption calculation (uplink + downlink)

Table 2 shows the values used for the simulation parameters.

The linear program was implemented in the Java programming language and solved using the IBM CPLEX solver 12.8 on a personal computer with Intel Core i7-5500U CPU, 2.40 GHz, 32 Gb RAM computer running the Linux Fedora operating system. The sensitivity of the solver’s optimality gap has been set to $1\times 10^{-7}$ to have enough precision to detect the optimal schedule length. Due to the complexity of the model, we have computed the optimal schedule only for network sizes between 10 to 50 nodes for fixed and variable node buffered data sizes. For the rest of the simulations, we vary the number of nodes from 100 to 1000 and we measure the data collection time, the packet delivery ratio (PDR), and the energy consumption of the nodes. We generate 50 instances per simulation run and the average results are presented along with the 95% confidence intervals.

The evaluation was conducted considering fixed as well as variable buffered data sizes. In the first use case, all nodes have the same amount of data to transmit (∼10 kB), which represents the buffered data per certain period of time from a periodic data generation application. This use case is a typical scenario in precision agriculture or environmental monitoring applications where the nodes periodically wake up, take a measurement, and then return to sleep mode. In the second use case, the nodes have a different amount of data to deliver whose size varies between 200 bytes and 18 kB (i.e., 2 to 180 transmissions). This use case represents data generation that is triggered by an event whose detection wakes up nodes to capture the event.

We would also like to stress that for the sake of simplicity as well as of energy efficiency all the packet sizes of all SFs are fixed and large enough. As it has been shown [16], it is more energy efficient to use large packet sizes for all SFs to perform the data collection. This is due to the increased overhead that small packet sizes cause at the MAC layer, thus, delaying the data collection process and increasing energy consumption. On the other hand, in a network with high error rate, very large packet size would cause a high loss of data or high energy cost in case of re-transmissions. Hence, in this work a packet size in the midpoint between the lowest (8 bytes) and the highest (255 bytes) packet size is chosen.

### 6.2. Scheduling Comparison with the Optimal

**Figure 4:**
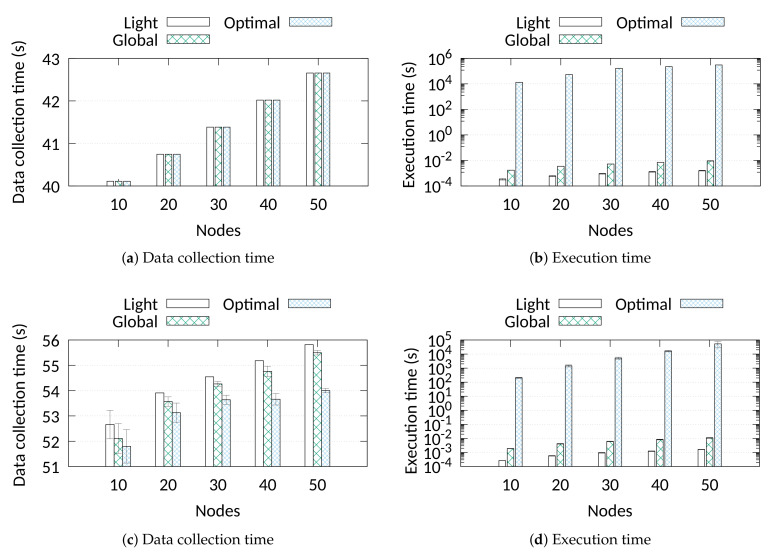
Comparison with the optimal for a scenario with fixed data size (upper) and variable data size (lower).

In this subsection, we compare the pure algorithmic performance of the scheduling approaches, without considering network requirements such as sync packets. We compare the performance between two heuristics, *Light* and *Global*, and the optimum solution as it is computed by solving the optimisation problem defined in Section 5. As we can observe from Figure 4a, both heuristics achieve identical performance to the optimum in terms of data collection time for all node populations. Moreover, this performance is achieved with a very small computation time which does not exceed 1.7 ms for *Light* and 9.9 ms for *Global* (see Figure 4b). On the contrary, due to the increased range of binary values of some parameters, such as the number of slots, the solver may require multiple hours to find the optimal solution.

We repeat the comparison using variable data sizes randomly assigned to the nodes in the range 200 bytes to 18 kB. This performance evaluation is depicted in Figure 4c,d. The results reveal that for the tested scenarios the two heuristics perform only up to 3% worse compared to the optimal solution. As it was expected *Global* achieves a slightly better performance than *Light* in terms of data collection time. The gap occurs as *Light* is unable to assign consecutive node transmissions to different—and eventually more efficient—frames, since it schedules only the first transmission. Finally, similarly to the fixed data size scenario, the transmission schedules are calculated in a very short time.

### 6.3. Comparison with Other Approaches

#### 6.3.1. Fixed Data Sizes

**Figure 5:**
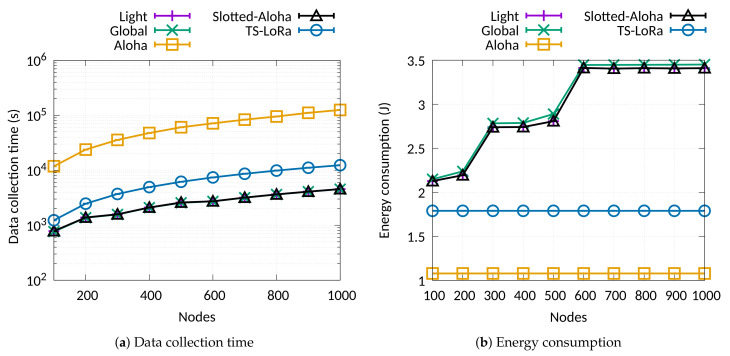
Data collection time and energy consumption for a scenario with fixed data size.

Simulation results with node numbers from 100 to 1000 are presented in Figure 5. The results do not include the schedule transmission overhead, however, this is almost negligible compared to the total values. The overhead varies from 3% to 7% (worst case) of the total data collection time and energy consumption. As we can observe, the impact of the collision-free transmissions in the data collection time is obvious. The performance in terms of data collection time is at least 10 times higher using an Aloha MAC approach such as LoRaWAN. Slotted-Aloha performs better, however, still far from the collision-free method.

In our simulations, all the approaches achieved a packet delivery ratio over 90% while the collision-free ones exceeded 95%. The Aloha approaches achieved those PDR values only at the expense of much longer data collection times. This shows the superiority of collision-free time-slotted communications as the number of nodes increases. Moreover, the scheduling of transmissions over different slots and SFs leads to much lower data collection time compared to TS-LoRa (non-scheduling approach). The improvement seems to be higher when the number of nodes increases. However, in terms of energy consumption, this action causes an increased trend in energy cost. This happens due to two reasons. Firstly, as the number of nodes increases, more and more nodes are switched to higher SFs. Higher SFs cause longer transmission times and, thus, higher energy consumption. Secondly, the nodes periodically turn on their radio to receive the sync packet from the gateway, increasing the average consumption during a round. Comparing TS-LoRa with the Pure Aloha MAC performance, we can see that the synchronisation overhead causes about 15% higher energy consumption (see Figure 5b). However, as we showed in one of our recent studies [31], the synchronisation cost is much lower than that caused by re-transmissions in an Aloha-based protocol.

#### 6.3.2. Variable Data Sizes

**Figure 6:**
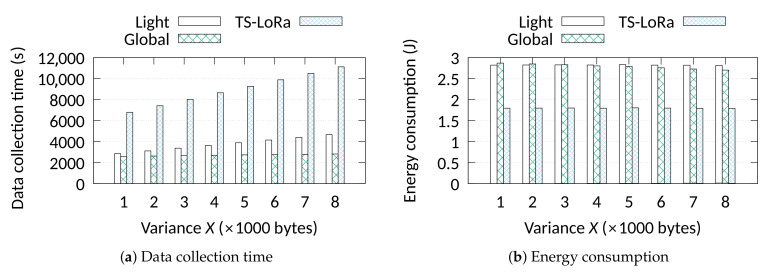
Data collection time and energy consumption for variable data sizes.

In the last set of simulations (see Figure 6), we focus on the comparison between the synchronous approaches in a variable data size scenario. In this scenario, 500 nodes are used where the data size of each node is randomly allocated from [10,000 −X, 10,000 +X] bytes, where X=[1000,...,8000]. The Aloha approaches are not included in this study for presentation purposes due their very high data collection times. *Global*’s advantage is obvious due to its capability to fill the empty slots left by nodes that finish transmissions earlier than nodes with more transmissions. *Light* schedules nodes’ activities based on the first transmission without considering the amount of data per node. As a consequence, nodes that complete sooner leave empty and never again used slots. TS-LoRa provides no SF re-assignment resulting in very high frame sizes. The performance gap increases with higher data variance since *Global* finds more options to fill empty slots. We can see that *Global*’s performance is almost independent of the data variance, while *Light*’s and TS-LoRa’s ones present an increasing trend. In terms of energy consumption, the difference between TS-LoRa and the scheduling approaches is about 45%. All the approaches have the same synchronisation cost per frame but the scheduling approaches spend more energy due to the higher average SF.

## 7. Proof of Concept and Experimental Results

### 7.1. System Setup and Procedure

**Figure 7:**
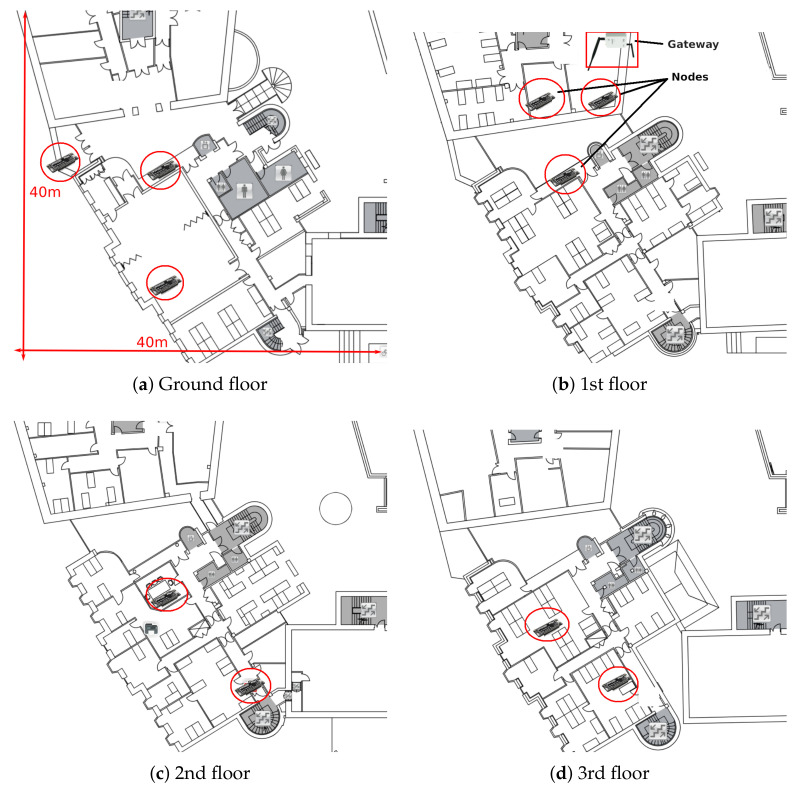
Positions of the nodes and the gateway in the 4-floor building.

In order to verify our approach also in experiments with real node deployments, we created a proof-of-concept implementation consisting of 10 nodes located across two adjacent, multi-floor buildings. Independent, co-located LoRaWAN networks also existed in the area. The top view with indicative node positions is illustrated in Figure 7. The gateway is located at the first floor on the edge of the upper-hand building. The maximum (reliable) indoor range that was achieved with SF7 was approximately 60 m using typical 2 dBi antennas for both the nodes and the gateway. This low range is justified by the presence of multiple concrete and stone walls which cause a very strong signal attenuation. The purpose of the experiments is not to show the superiority of the scheduling approaches over the rest of the methods since this would require a network of at least 100 nodes along with very high financial and time resources. Thus, we limit the purpose of the experiments to assess the following practical issues:

1. to assess the feasibility of using time-slotted scheduling algorithms under real conditions,
2. to assess the downlink transmission time of the schedule,
3. to assess the feasibility of computing the schedule on the nodes instead of computing it centrally on the gateway and then disseminating it to the nodes, and
4. to assess confirmed transmissions.

LoRa-enabled devices from Pycom in the Netherlands where used for the purpose of the experiments. For experimental efficiency, an amount of data was already generated and loaded onto the nodes’ storage module. Each node position was initially evaluated for its connectivity requirements in terms of transmit power and SF. We did this test manually, however LoRaWAN’s ADR mechanism can be used to automatically calculate and adjust the SF according to the latest conditions (the embedded code of the gateway and the nodes is available at https://github.com/deltazita/offline-lora (accessed on 3 February 2021) and at https://github.com/deltazita/ts-lora (accessed on 3 February 2021)). Table 3 summarises the experimental parameters and their corresponding values.

**Figure 8:**
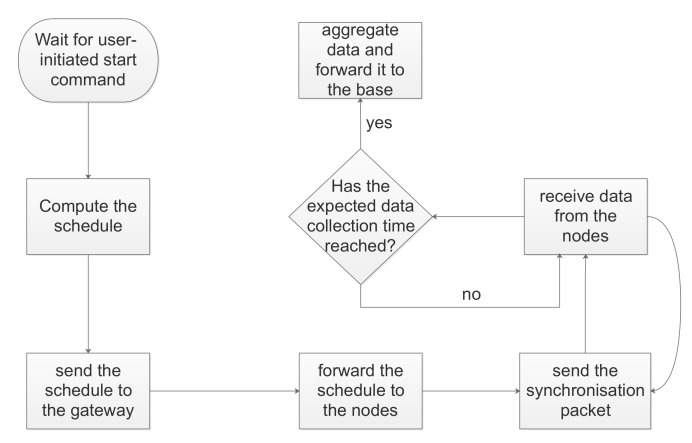
Experimental procedure of Base/Gateway.

**Figure 9:**
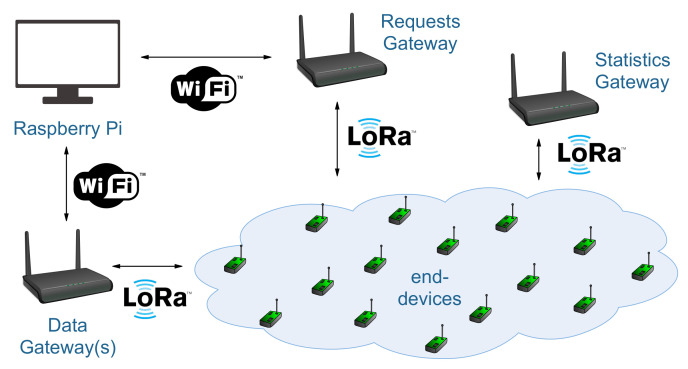
Network architecture of the experiments.

The entire process of computing the schedule, communicating it to the nodes, and receiving the data is illustrated in Figure 8. Moreover, an overview of the network architecture is presented in Figure 9. We used a Raspberry Pi (here representing the network server) to compute the schedule. The Raspberry Pi can also optionally send additional information related to the experimental process, such as the guard time, the synchronisation rate, and the channel bandwidth. A gateway is responsible for registering nodes to the network, while another gateway is used for data collection. A third gateway can optionally be used for statistic collection in case of confirmed transmissions (i.e., the nodes can send out data about the ack rate). All the gateways communicate with the Raspberry Pi via secure WiFi links. The data gateway receives the schedule and forwards it to the nodes. To do so, the nodes must have their radio on and listen to a specific channel, BW, and SF. These settings are fixed and do not change throughout the process, thus, they can be flashed into the nodes during their setup by a system administrator. In the experiments, we used the highest SF in the network so that all nodes regardless of their SF can receive the schedule. An alternative approach would be to send the schedule multiple times based on the available SFs in the network. However, this would require additional setup time and probably to a waste of energy for the nodes, unless multiple LoRa gateways were used (one gateway for each available SF).

We run each experiment 10 times and the average results are presented along with the minimum and the maximum values. We measure the schedule transmission time, the data collection time, the active radio time and the packet delivery ratio for the 10-node testbed.

Data transmissions are performed in the allocated time slots using the assigned SFs as computed by the scheduler. The nodes have their radio off during the rest of the period, however, they periodically need to turn on their radio to receive synchronisation packets from the gateway. The synchronisation is achieved using an additional slot at the end of a frame as it was presented in Section 4.1. The reader can find more information about this mechanism in [31].

### 7.2. Transmission of the Schedule

Here, we present experimental results related to the transmission of the schedule. This includes an evaluation of the capability of the gateway to efficiently compute and encode the schedule. No physical presence of the nodes themselves is required. As a consequence, we can experiment with more than 10 nodes.

In *Light*, the schedule consists of a set of tuples indicating the node id, the SF, and the slot number (i.e., [id, sf, slot]). For example, in our 10-node deployment this results in a 70 byte schedule. Some extra bytes are added to indicate the gateway id, the guard time, and the sync packet rate. In total, 78 bytes are sent. In terms of transmission time this translates to 35 ms with SF7 or 698 ms with SF12.

*Global* uses a more sophisticated data encoding to represent the repetition of the frames in the schedule. Recall that in *Global* all the packets are scheduled, thus, a collection of frames is generated. A straightforward solution would be to include the node ids, their SFs, and their slots for all the computed frames in the schedule. However, this solution does not scale because the number of bytes increases linearly with the number of transmissions. On the contrary, since a big range of successive transmissions is scheduled on the same SF and slot, one way to reduce the total number of bytes is to use a run-length encoding data compression algorithm. For example, in our testbed, only the last transmission of one node is relocated to SF8. Thus, all the previous transmissions can be indicated using a single tuple. The general encoding pattern of a node’s transmissions is the following: [id first_frame_sf last_frame_sf sf slot, start_frame_sf’ end_frame_sf’ sf’ slot’, ...]. In our experiments, the total schedule length with *Global* is 154 bytes. In terms of transmission time this translates to 6.3 ms and 1.23 s with SF7 and SF12, respectively.

Every node that receives the schedule, decodes the data and calculates the frame length for its assigned SF. In *Global*, this must be done for all the assigned SFs which incurs additional coding effort. The schedule transmission is followed by a very short init packet which actually initiates the data collection process. In the Aloha-based approach, the schedule transmission is omitted, thus, only the init packet is used.

Table 4 illustrates the total theoretical schedule transmission time for different node populations using SF7. We examine the scenario where the schedule is sent based on an ideal setup where no duty cycle restrictions are imposed as well as when the duty cycle rules are followed. We should note that the first case occurs when transmissions are sent in non-restricted bands (e.g., 2.4 GHz LoRa). We can observe that, in the first case, the transmission of the schedule is quite fast for up to 100 nodes. *Light* scales very well with less than 4 s transmission time for 1000 nodes. On the contrary, as the number of nodes increases, *Global*’s schedule becomes very long and, thus, difficult to be transmitted in a reasonable period of time, especially when the duty cycle rules have to be followed. This is because each node’s transmissions may jump from one SF to another causing many non-periodic slot allocations in successive frames. Letting the nodes know these SF jumps, requires extra bytes and, thus, more time.

To overcome the scalability issue of *Global*, we follow an alternative approach [11]. We disengage the computation of the schedule from the gateway (network server) and we move this process to the nodes. In this way, we transmit only the list of nodes and their minimum SFs, and we let the nodes compute the schedule. Since this list is usually multiple times shorter in size than the schedule, it can be transmitted in a shorter amount of time. It should be noted that this action leads to a trade-off between processing and communication time. In order to evaluate this trade-off, we implement *Global* on our testbed devices. Due to the limited memory resources (only 4 MB), the nodes compute the entire schedule but they generate a list of transmissions (i.e., slots and SFs) only for their own id. The input transmission time, as well as the schedule computation time, are displayed in Table 5. The results show an up to 78% improvement in total transmission time in the case without duty cycle restrictions and an over 800% improvement when the duty cycle rules are followed. We also need to stress here that the energy consumption in processing mode is comparable to the receiving cost (i.e., ∼50 mA), so the nodes do not spend more energy compared to when the schedule was transmitted by the gateway.

### 7.3. Confirmed vs. Non-Confirmed Traffic

**Figure 10:**
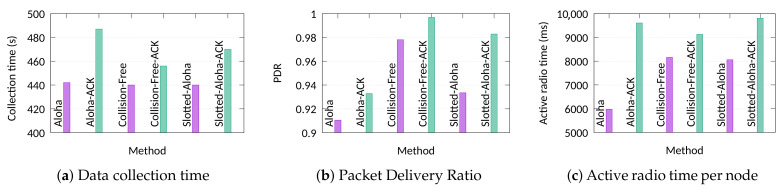
Experiment with 10 nodes: Data collection time, Packet Delivery Ratio, and active radio time with and without retransmissions (Collision-Free approaches, Slotted-Aloha, -ACK: the corresponding approach with retransmissions).

Finally, in the last experiment, we assess the behaviour of the approaches when re-transmissions can occur. For the needs of these experiments, we also developed approaches that allow re-transmissions of packets in order to capture the effect of acknowledgments in the data collection time and in other measurements. All the nodes transmit 100 unique packets, while maximum 3 retransmissions per packet are allowed before the packet gets dropped. For the time-slotted approaches (i.e., Slotted-Aloha, *Light*, *Global*, and TS-LoRa) the acknowledgments are sent in groups during the SACK packet. The periodicity of the SACK packet is communicated to the nodes at the beginning of the process. In the Pure Aloha approach, acknowledgements are sent similarly to LoRaWAN, using two receive windows. Figure 10 depicts the average data collection time, the PDR, and the active radio time for the experiments. Due to the low number of nodes, all collision-free approaches presented identical results. Thus, the label “CF” refers to all these approaches, while the label “CF-ACK” refers to their confirmed version. Moreover, we must mention that the two heuristic scheduling approaches were able to generate optimal schedules. The results reveal that in terms of data collection time, there is a 10% overhead due to synchronisation compared to the optimal solution. This overhead is apparently higher in case of re-transmissions. The two Aloha approaches present the highest overhead due to higher number of collisions and, thus, more time is required because of the re-transmissions. As it was expected, the PDR is very high for the collision-free approaches and ranges from 98% to 99.8%. Because no intra-network collisions occur, some packets were lost due to the path-loss effects and the presence of external interference. Slotted-Aloha performs close enough in confirmed traffic. This is because in our implementation acknowledgements are sent straight away after the reception of packet (unlike LoRaWAN where 1 or 2 s delay is imposed). Finally, as it was expected, the slotted approaches exhibit a higher energy cost compared to Pure Aloha without acknowledgements but they present a better performance than Aloha in the case of confirmed transmissions. The reason that this occurs is that in the frame structure of TS-LoRa (which is also followed by *Light* and *Global*), the acknowledgements are sent in groups at the end of the slot. This minimizes the number of bytes received by the nodes and, thus, their energy consumption.

## 8. Conclusions and Future Directions

In this work, we studied the problem of bulk data collections in LoRa-based networks for situations where a gateway is not available to receive data at all times due to the lack of a permanent power source or the lack of coverage (e.g., in satellite IoT systems). We showed that traditional Aloha-based networks suffer from very long data collection times in order to achieve a satisfactory data delivery ratio. Here, we explored the possibility of using collision-free time-slotted LoRa communications together with SF and slot scheduling to accelerate the data collection. We mathematically formulated and solved the corresponding scheduling problem of finding the best SF and slot allocations that optimise the data collection time. The comparison with heuristics for small data sets showed only a slight performance gap in data collection time compared to the optimum approach. We implemented our methods using real deployments and we examined critical components of the bulk data collection such as the transmission of the schedule. Despite the duty cycle and power limitations, the results demonstrated the significant advantage of using the proposed synchronous approaches to achieve fast data collection especially with hundreds of nodes.

For future work, we intend to investigate the possibility of using medium sensing techniques for data offloading in dense node situations and see their effect on the data collection time. More specifically, we intend to assess the use of the Channel Activity Detection (CAD) mechanism of LoRa transceivers in scenarios where a high packet rate is required, and also develop a feedback and re-transmission mechanism for those scenarios. Another interesting problem to explore is the transmission of the schedule using multiple gateways. We are planning to design an approach to divide the schedule in multiple pieces and transmit it to the nodes over different channels and SFs in order to accelerate the process and increase the levels of scalability—but also of energy efficiency—in large-scale networks. Finally, an open problem remains the development of a payload-agnostic time-slotted algorithm for data collection purposes.

## Figures and Tables

**Table 1:** Notations and their meaning.

Notations	Meaning
*f*	Spreading Factor (SF) value
$N_{f}$	Number of nodes with SF *f*
$T_{f}$	Transmission time with SF *f*
$payload_{f}$	Payload size per SF *f*
θ	Packet data rate
$\bar{L_{pl}}\left(\right)$, $d_{0}$, γ, σ	Path-loss model parameters
$P_{f}^{min}$	Minimum success probability for SF *f*
$P_{given}$	Minimum required success probability
*C*	Radio duty cycle constraint (0–1)
*F*	Set of available SFs
$F_{i}$	Set of possible SFs per node *i*
$pkt_{i}$	Number of transmit packets per node *i*
τ	Guard time length
Γ	Upper limit in number of slots in a frame
$T_{f}$	Number of slots in frame for SF *f*

**Table 2:** Simulation Parameters.

Parameter	Value
Nodes	100–1000
(comparison with the model)	10–50
Terrain side	1000 m
Gateway coordinates	(500 m, 500 m)
Channel bandwidth (BW)	500 kHz
Preamble Symbols	8
Coding Rate	4/5
Payload size	100 bytes
Buffered Data per node	10,000 bytes (unless specified)
(comparison with the model)	1000 bytes or 500–1500
Path Loss model (see [6])	$\bar{L_{pl}}\left(d_{0}\right)=95$ dB (experimental)
	$d_{0}=40$ m, γ=2.08, σ=3.57
Guard time (τ)	10 ms for up to 50 nodes,
	40 ms otherwise
Receiver Sensitivities	[−116, −119, −122, −125,
(per SF for BW500)	−128, −129] dBm
Tx power & consumption	14 dBm, 75 mA [31]
Rx consumption	45 mA [31]
Aloha transmission rate ($\theta_{max}$)	derived from Equations (2)–(5)
Radio duty cycle (*C*)	≤1%

**Table 3:** Experimental parameters.

Parameter	Value
Nodes	10
Channel bandwidth	500 kHz
Preamble Symbols	8
Coding Rate	4/5
Schedule size	78 bytes (*Light* algorithm)
	156 bytes (*Global* algorithm)
Packet size	100 bytes (98 bytes for data and 2 bytes overhead)
Sync packet size	8 bytes
Data per node	9800 bytes
Guard time	15 ms
Tx power (nodes)	14 dBm
Pure & Slotted Aloha packet rate	∼13.5 *pkt*/min with SF7
	(duty cycle bounded)
Max retransmissions	up to 3
Radio duty cycle (nodes)	≤1%
Radio duty cycle (gateway)	≤10%

**Table 4:** Schedule transmission times following or not following the duty cycle rules (seconds).

Nodes	No Duty Cycle		Duty Cycle	
	*Light*	*Global*	*Light*	*Global*
10	0.035	0.063	0.035	0.063
100	0.389	1.362	29.3	126.64
500	1.85	223.31	175.32	>1000
1000	3.796	490.03	370.01	>1000

**Table 5:** Schedule computation time by the nodes and transmission time of the input using *Global* (seconds).

Nodes	Input (No Duty Cycle)	Input (Duty Cycle)	Processing
10	0.027	0.027	0.77
100	0.29	19.56	8.49
500	1.17	107.1	47.36
1000	2.34	223.98	108.03

## Data Availability

Not applicable.
